# Oil Palm Research in Context: Identifying the Need for Biodiversity Assessment

**DOI:** 10.1371/journal.pone.0001572

**Published:** 2008-02-13

**Authors:** Edgar C. Turner, Jake L. Snaddon, Tom M. Fayle, William A. Foster

**Affiliations:** Insect Ecology Group, University Museum of Zoology, Cambridge, United Kingdom; University of Zurich, Switzerland

## Abstract

Oil palm cultivation is frequently cited as a major threat to tropical biodiversity as it is centered on some of the world's most biodiverse regions. In this report, Web of Science was used to find papers on oil palm published since 1970, which were assigned to different subject categories to visualize their research focus. Recent years have seen a broadening in the scope of research, with a slight growth in publications on the environment and a dramatic increase in those on biofuel. Despite this, less than 1% of publications are related to biodiversity and species conservation. In the context of global vegetable oil markets, palm oil and soyabean account for over 60% of production but are the subject of less than 10% of research. Much more work must be done to establish the impacts of habitat conversion to oil palm plantation on biodiversity. Results from such studies are crucial for informing conservation strategies and ensuring sustainable management of plantations.

## Introduction

**Figure 1:**
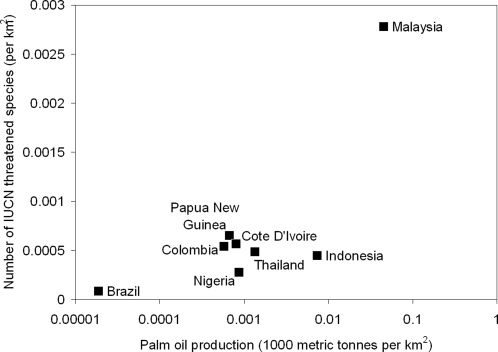
Number of threatened species per km^2^ (IUCN Red List of Threatened Species [27]) in relation to palm oil production per km^2^ in 2005 [2] in the top eight palm oil producing countries.

The expansion in oil palm (*Elaeis guineensis*) cultivation is frequently cited as being a major factor driving deforestation and biodiversity loss in tropical countries [1]–[3]. The area under oil palm has increased dramatically in recent decades and covers over 10.7 million hectares worldwide; an increase of 168% since 1960 [1]. Oil palm-derived oil is now the world's major source of vegetable oil and fat, with over 37 million metric tons produced in 2005, around 27% of the total global production. The oil is ubiquitous in the food industry as well as the oleochemical industry, where it is used for making soaps and detergents. Oil palm is a tropical crop and is cultivated in lowland areas from South America to Africa and Asia. Malaysia and Indonesia are the leading producers of palm oil, exporting 15.0 and 14.1 million metric tons respectively in 2005 [2]. Oil palm production is therefore centered on highly biodiverse regions with high levels of endemism [3]–[5]. Higher levels of palm oil production are also generally associated with a higher number of endangered species. Malaysia has by far the highest levels of palm oil production per unit area and the highest relative number of endangered species (Fig. 1). Recent decades have seen a diversification in the uses of palm oil, for example in feed for livestock and fisheries. Alternative uses for the oil and for byproducts of the plantation system are also being investigated, with interest focusing on their potential as a source of biofuel. With the heightened demand for palm oil as its uses expand, oil palm production is set to increase in the future [2], [6]. Potentially this has extensive negative consequences for biodiversity in these areas. Here we investigate whether sufficient research is being done on the impacts of oil palm cultivation on ecosystems. We find that while the focus of oil palm research has changed over the last thirty years, far more still needs to be carried out to quantify the impacts of this important crop on biodiversity.

## Methods

ISI Web of Science (WoS) [7] was used to investigate how research into oil palm has varied as its cultivation has increased. We used the search term ““palm oil” or “oil palm”” and viewed the resulting 3056 publications between 1970 and 2006, recording the number found and classifying them according to their main research focus as interpreted through their title, abstract, key words, journal title and subject classification. It was therefore possible to visualize how the focus of oil palm research has changed since 1970. The number of oil palm publications was compared to the number of publications on agriculture (search term: “agriculture”) and to global palm oil production. We also compared the number of publications across the main vegetable oil crops (search term: ““vegetable crop name” and “oil””) with global oil production.

## Results and Discussion

**Figure 2:**
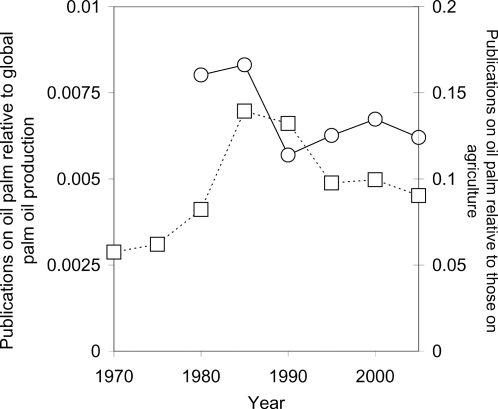
Relative number of publications on oil palm between 1970 and 2005. Round symbols show publications relative to palm oil production worldwide between 1980 and 2005 [2]. Square symbols show publications relative to those on agriculture between 1970 and 2005 [7].

**Figure 3:**
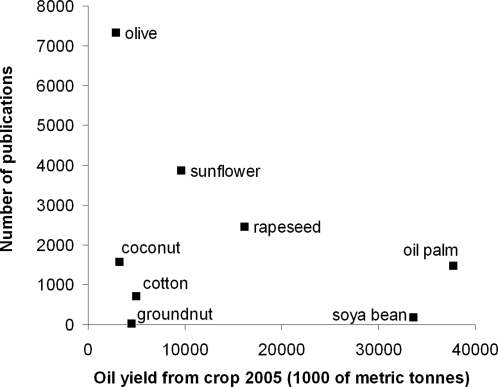
Oil yield from top eight crops in 2005 [2] in relation to total number of publications on each crop. Publication frequency between 1970 and 2006 was assessed using Web of Science [7].

**Figure 4:**
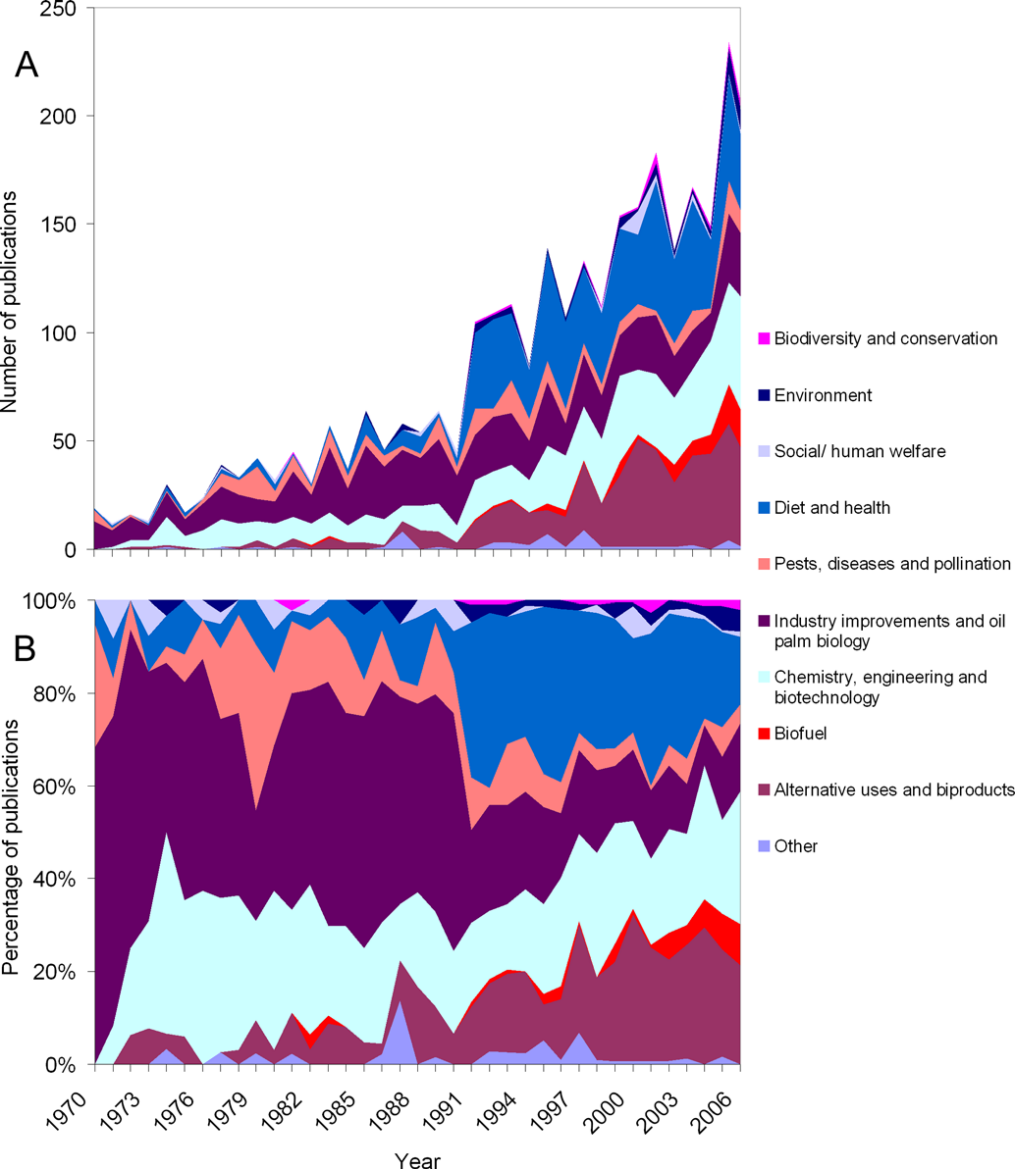
Changes in the focus of research on oil palm between 1970 and 2006 [7]. Results are plotted as (A) total number of publications and (B) percentage of publications.

The number of oil palm publications relative to those on agriculture has fluctuated considerably over time but has shown a net increase. In contrast, the number of publications relative to oil palm production has shown a net decrease (Fig 2). Despite being a major source of vegetable oil worldwide, oil palm has attracted relatively little research interest compared to other oil crops. Palm oil and soyabean contribute over 60% to the world's vegetable oil production but have less than 10% of the research interest (Fig. 3). Over the last 35 years, the major focus of oil palm research has been its uses in food and the resultant health issues (22.19% of total, 678 publications). In the last ten years there has been a marked increase in the number of publications on i) byproducts from the oil palm industry, ii) chemistry, engineering and biotechnology, and iii) the production of biofuel. The number of publications on biodiversity (0.75% of total, 23 publications) and other environmental issues (2.06% of total, 63 publications) has been extremely low (Fig. 4).

Publications concentrating on biodiversity and species conservation have tended to focus on large animals [8]–[10] and birds [11], [12] where a severe negative impact on biodiversity has been noted [13]. Although these larger animals are important flagships for the state of the tropical environment, they are not good indicators of oil palm plantation biodiversity. The vast majority of species worldwide are insects, which carry out the lion's share of the ecosystem function [14]. We found only five publications that relate to the impact of conversion of forest to oil palm plantations on invertebrate biodiversity [15]–[19].

Understanding the impacts of oil palm expansion on invertebrates and other taxa is vital given the projected increase in oil palm area. Not only will such information allow us to make informed judgments as to the genuine status of biodiversity in oil palm plantations, but it will also allow us to begin to quantify how well these managed ecosystems are functioning. By understanding how different taxa and guilds are affected by oil palm expansion we can begin to see how management can be manipulated to enhance beneficial ecosystem functions with minimum detrimental effects on productivity. Agricultural theory highlights the use of integrated pest management in plantation and agricultural systems [20]–[22]. Such management draws the link between components of the biotic environment and beneficial functioning in the managed landscape, and therefore may increase the yield or profit from an area whilst minimizing cost to biodiversity. In oil palm, much research has focused on the uses of focal species for pollination (e.g. the pollinating weevil *Elaeidobius kamerunicus*, [23]) or parasitization of herbivores [21], [22]. We suggest that the focus now needs to be on the function of biological diversity in the plantation. Can practices to increase biodiversity be integrated with traditional management to produce a net benefit for plantation managers and the environment alike? Management for the benefit of a wider range of species increases the stability of ecosystem functions [24]. This is likely to lead to greater sustainability in the future as ecosystem function is less tied to the fortunes of individual species.

Management to enhance biodiversity may not be as costly in terms of yield or profit as it may first appear, as management techniques may provide other benefits. For example, maintenance of forest fragments in the plantation landscape is likely to increase biodiversity, but is also likely to reduce erosion and flooding if maintained around river margins. Flooding can be a major cause of mortality in oil palms and incur huge costs. Recent publications have also pointed out that oil palm estates are effectively self-sufficient villages [3]. Therefore maintaining biodiversity in a plantation context is also potentially important from recreational and educational points of view, as oil palm has already become the countryside for a large number of people in the tropics. Without an awareness of biodiversity, future generations are less likely to value and protect it [25].

Despite the lack of research on the subject, several groups have formed in the last few years aiming to promote and investigate the potential of sustainable palm oil cultivation [13]. The most influential of these is the Roundtable on Sustainable Palm Oil (RSPO), which has made considerable progress in promoting sustainable plantation management [2], [26]. It is now up to ecologists to provide information on the biodiversity status of oil palm plantations and to investigate practices that may benefit biodiversity, the environment, the community and the industry alike.
